# Molecular Features of Polycystic Ovary Syndrome Revealed by Transcriptome Analysis of Oocytes and Cumulus Cells

**DOI:** 10.3389/fcell.2021.735684

**Published:** 2021-09-06

**Authors:** Jie Li, Haixia Chen, Mo Gou, Chenglei Tian, Huasong Wang, Xueru Song, David L. Keefe, Xiaohong Bai, Lin Liu

**Affiliations:** ^1^The State Key Laboratory of Medicinal Chemical Biology, Department of Cell Biology and Genetics, College of Life Sciences, Nankai University, Tianjin, China; ^2^The Center for Reproductive Medicine, Tianjin Medical University General Hospital, Tianjin, China; ^3^Department of Obstetrics and Gynecology, NYU Langone Medical Center, New York, NY, United States

**Keywords:** PCOS, transcriptome, oocytes, cumulus cells, microtubule, ERV

## Abstract

Polycystic ovary syndrome (PCOS) is typically characterized by a polycystic ovarian morphology, hyperandrogenism, ovulatory dysfunction, and infertility. Furthermore, PCOS patients undergoing ovarian stimulation have more oocytes; however, the poor quality of oocytes leads to lower fertilization and implantation rates, decreased pregnancy rates, and increased miscarriage rates. The complex molecular mechanisms underlying PCOS and the poor quality of oocytes remain to be elucidated. We obtained matched oocytes and cumulus cells (CCs) from PCOS patients, compared them with age-matched controls, and performed RNA sequencing analysis to explore the transcriptional characteristics of their oocytes and CCs. Moreover, we validated our newly confirmed candidate genes for PCOS by immunofluorescence. Unsupervised clustering analysis showed that the overall global gene expression patterns and transposable element (TE) expression profiles of PCOS patients tightly clustered together, clearly distinct from those of controls. Abnormalities in functionally important pathways are found in PCOS oocytes. Notably, genes involved in microtubule processes, *TUBB8* and *TUBA1C*, are overexpressed in PCOS oocytes. The metabolic and oxidative phosphorylation pathways are also dysregulated in both oocytes and CCs from PCOS patients. Moreover, in oocytes, differentially expressed TEs are not uniformly dispersed in human chromosomes. Endogenous retrovirus 1 (*ERV1*) elements located on chromosomes 2, 3, 4, and 5 are rather highly upregulated. Interestingly, these correlate with the most highly expressed protein-coding genes, including tubulin-associated genes *TUBA1C*, *TUBB8P8*, and *TUBB8*, linking the *ERV1* elements to the occurrence of PCOS. Our comprehensive analysis of gene expression in oocytes and CCs, including TE expression, revealed the specific molecular features of PCOS. The aberrantly elevated expression of *TUBB8* and *TUBA1C* and *ERV1* provides additional markers for PCOS and may contribute to the compromised oocyte developmental competence in PCOS patients. Our findings may also have implications for treatment strategies to improve oocyte maturation and the pregnancy outcomes for women with PCOS.

## Introduction

Polycystic ovary syndrome (PCOS) is the most common endocrinopathy in women of reproductive age, with a prevalence of about 10% (March et al., 2010). The syndrome is typically characterized by a polycystic ovarian morphology, hyperandrogenism, and ovulatory dysfunction. Additional clinical features include insulin resistance, obesity, type 2 diabetes (T2D), and infertility (Azziz et al., 2016). Furthermore, a recent study shows that daughters of women with PCOS are more often diagnosed with PCOS. PCOS phenotype can also be transgenerationally transmitted across offspring of female mice (Risal et al., 2019). Previous genome-wide association study (GWAS) analysis identified 11 loci associated with PCOS and candidate genes at these loci, which were related to the clinical manifestations of PCOS, such as infertility, insulin resistance, T2D, and others (Chen et al., 2011; Shi et al., 2012). These studies facilitated an understanding of the etiologic factors accounting for PCOS. Microarray or RNA sequencing analysis of oocytes and/or granulosa cells or cumulus cells (CCs) in women with PCOS has provided insights into the understanding of PCOS (Wood et al., 2007; Haouzi et al., 2012; Wissing et al., 2014; Liu et al., 2016; Chen et al., 2019). In addition, studies focused on ovarian somatic cells or ovary and other tissues revealed the metabolic abnormalities in PCOS (Ma et al., 2007; Corton et al., 2008; Huang et al., 2010; Zhao et al., 2017; Sanchez-Garrido and Tena-Sempere, 2020). The status of metabolism in the oocytes of PCOS patients remains elusive.

Notably, at least 40% of the human genome derives from transposable elements (TEs), yet the transcription of TEs in PCOS patients remains to be comprehensively investigated. TEs are categorized into two features – DNA transposons accounting for 3% of TEs and retrotransposons representing 90% of TEs (Friedli and Trono, 2015), and the retrotransposons are further divided into five orders including long terminal repeat (LTR), *Dictyostelium* intermediate repeat sequence (DIRS), Penelope-like element (PLE), long interspersed nuclear element (LINE), and short interspersed nuclear element (SINE) (Wicker et al., 2007). Endogenous retroviruses (ERVs), as a superfamily of LTR retrotransposons, can copy and paste their own DNA into the genome (Bannert and Kurth, 2006; Cordaux and Batzer, 2009). TEs also play essential roles in transcriptional modulation, and specific ERV families are transcribed during human preimplantation development, which is stage specific (Goke et al., 2015). Interestingly, a subset of ERV1s and of ERVKs are associated with meiotic gene expression and act as enhancers to activate meiotic genes in human spermatogenesis (Sakashita et al., 2020). Somatic retrotransposons can alter the expression of protein-coding genes differentially expressed in the human brain (Baillie et al., 2011). Malki et al. (2014) proposed that high levels of L1 (LINE 1) link to enhanced oocyte elimination, and L1 activity may be involved in controlling the size and the quality of mammalian ovarian oocyte reserves, yet the L1 methylation levels are only slightly changed in CCs of oocytes from patients with PCOS (Pruksananonda et al., 2016). Dysregulated TEs are involved in gene mutation and the occurrence of a number of human diseases, including malignancies, neurological disease, and normal aging (Payer and Burns, 2019).

In this study, we systematically analyzed TE and global gene expression in oocytes and CCs from PCOS patients and compared them with controls. We identified new candidate genes and TEs underlying PCOS, which may serve as biomarkers of PCOS.

## Materials and Methods

### Human Subjects

The study subjects included five women without PCOS (controls) with body mass index (BMI) between 17.70 and 23.50 kg/m^2^ and five PCOS patients with BMI between 19.00 and 28.10 kg/m (*P* = 0.114). The average age of the PCOS patients and controls was 32.40 ± 1.29 and 35.60 ± 2.23, respectively (*P* = 0.249). The demographic and clinical characteristics of all participants, including age, BMI, LH, and others, were collected and summarized in Supplementary Table 1.

The PCOS patients were diagnosed according to the Rotterdam criteria (Rotterdam, 2004a,b), which meets two of the following three features: oligo- or anovulation, clinical and/or biochemical signs of hyperandrogenism, and polycystic ovary by ultrasound. We excluded other etiologies, such as congenital adrenal hyperplasia, androgen-secreting tumors, and Cushing’s syndrome. Women included in the control group had regular menstrual cycles, normal sonographic appearance of ovaries, and no diabetes or clinical signs of PCOS.

Informed consents were obtained from all the participants included in the study. The study was approved by the Ethics Committee of the Tianjin Medical University General Hospital (No.: IRB2018-102-01) and conducted in accordance with approved institutional guidelines.

### Ovarian Stimulation and Oocyte Retrieval

All participants underwent controlled ovarian stimulation using the GnRH antagonist protocols with the recombinant human follicle-stimulating hormone (rhFSH) and intracytoplasmic sperm injection (ICSI) for male fertility. An ultrasound scan and serum estradiol assays were performed for monitoring the follicular size. When two or more follicles were at least 12 mm in diameter, 10,000 IU human chorionic gonadotropin was administered 36 h before oocyte retrieval. The amount and duration of rhFSH treatment were similar in both PCOS patients and controls, exhibiting no statistically significant difference (Supplementary Table 1).

### Isolation of Single Oocyte and CCs

Cumulus–oocyte complex was isolated via ultrasound-guided vaginal puncture and classified according to the oocyte nuclear maturation stage: germinal vesicle (GV), metaphase I (MI), and metaphase II (MII). We only collected GV-stage oocytes and the surrounding CCs for this study, whereas MII-stage oocytes were used for clinical fertilization.

The CCs were collected as previously described (Haouzi et al., 2012). Briefly, the CCs were mechanically stripped from oocytes shortly under stereomicroscopy prior to ICSI, and then isolated CCs were dispersed into a single cell with 0.03% hyaluronidase (H6254-500MG, Sigma-Aldrich) and resuspended three times using phosphate-buffered saline (PBS). The separated CCs were counted up to 500 cells and placed in lysate. Tyrode’s Acidic Solution (T1788-100ML, Sigma-Aldrich) was used to facilitate the stripping of the zona pellucida to produce naked oocytes. The oocytes were observed under a microscope to ensure the absence of contamination with CCs. The naked oocytes were carefully washed three times using PBS with 0.1% polyvinylpyrrolidone (P0930-50G, Sigma-Aldrich) to prevent sticking to handling tools or dishes and then placed in lysate.

Six oocytes were collected from five PCOS patients, and six oocytes were also collected from five controls without PCOS (detailed in Supplementary Table 2). Among them, two oocytes were collected from one PCOS patient or one control. Two CC samples were collected from two PCOS patients and two controls; thus, two biological replicates of CCs were obtained from the PCOS patient or control group. The oocytes and CCs from three PCOS patients and three controls were used for RNA-seq. The oocytes from two PCOS patients and two controls were used for subsequent immunofluorescence microscopy or *in vitro* maturation (Supplementary Table 2).

### Library Construction From Oocytes or CCs and Sequencing

Individual oocytes or CC samples (500 cells) were transferred into a lysis buffer quickly, and Smart-seq2 protocol (Picelli et al., 2014) was used to synthesize the cDNA for single-cell RNA-seq analysis. After reverse transcription of mRNA and amplification of cDNA, real-time quantitative polymerase chain reaction (qPCR) analysis was performed to check the quality of the cDNA libraries using a housekeeping gene, GAPDH. The variation in the expression of GAPDH was minimal, and the negative control did not detect any product (Supplementary Figure 1A). The RNA-Seq libraries were constructed by TruePrep DNA Library Prep Kit V2 for Illumina^®^ (TD503-02, Vazyme Biotech) following the instruction manual. Meanwhile, to ensure the accuracy and repeatability of the RNA-seq data, we performed a duplicate when we constructed a library for every oocyte and collected CC samples to match with the retrieval oocytes from two PCOS patients and two controls, so each donor had two CC samples for RNA-seq. We performed 14 cycles of PCR to amplify the cDNA library and simultaneously barcoded it. The final indexed libraries were pooled and sequenced on an Illumina HiSeq X10 platform with a 150-bp paired-end read length.

### RNA-Seq Data Processing and Analysis

For RNA-seq raw reads with low-quality bases, adapters were trimmed by Trimmomatic (Bolger et al., 2014) to obtain clean reads (parameters: -PE -phred33 -SLIDINGWINDOW:4:15 -LEADING:10 -TRAILING:3 -MINLEN:36). Clean reads were aligned to the UCSC human hg19 reference using the Hisat2 with default settings (Kim et al., 2015). The read counts of each gene annotated in RefGene were calculated by featureCounts with default parameters (Liao et al., 2014). The RNA-seq libraries of each oocyte and each CC sample were sequenced at an average depth of approximately 4.6 million reads per oocyte and 6.8 million per CC sample (Supplementary Figure 1B), and the average ratio mapped for each oocyte and each CC sample was 72.98 and 54.88%, respectively (Supplementary Figure 1C). The read counts were loaded into RStudio (v3.6.1). For the accuracy of the gene expression levels, only genes with transcripts per million (TPM) > 1 in at least one oocyte or CC sample were analyzed. DESeq2 (Love et al., 2014) was used to obtain the statistical significance of differentially expressed genes (DEGs) between PCOS and control. Only the genes with a fold change of log2 transformed larger than log_2_(1.5) and adjusted *P* value < 0.05 from DEseq2 results were considered to be differentially expressed. The adjusted *P* values were computed in DESeq2 using the Wald test, adjusted for multiple testing using the procedure of Benjamini and Hochberg (Hochberg and Benjamini, 1990). Gene ontology (GO) and Kyoto Encyclopedia of Genes and Genomes (KEGG) analysis of DEGs was performed using DAVID (v6.8) (Huang da et al., 2009), and only the enriched pathways that showed a *P* value < 0.05 were chosen as significant enrichment. The gene expression level in a sample was quantified as the TPM, which was calculated according to the following formula: $TPM_{ij}=\frac{C_{ij}/\mathrm{length}\mathrm{of}\mathrm{gene}i}{\sum_{i}C_{ij}/\mathrm{length}\mathrm{of}\mathrm{gene}i}\times 10^{6}$, where C*_*ij*_* was the count value of gene *i* in sample *j*. Genes with expression transformed to the TPM values were used for t-SNE dimension reduction by R package “Rtsne” and t-SNE map drawn using the R package “ggplot2.” Diagrams were generated by the TPM value of each gene that were plus two then log2-transformed.

### Transposable Element Analysis

Clean reads were aligned to the UCSC human hg19 reference by STAR with parameters “-winAnchorMultimapNmax 100” and “-outFilterMultimapNmax 100” (Jin et al., 2015). Based on the previous method (Ohtani et al., 2018), only the TEs mapping their distributions in intergenic regions were considered, excluding the location between the transcription start sites and transcription end sites of genes. TEs annotated in UCSC Genome Browser (RepeatMasker) were counted using featureCounts. The reads mapped to TEs of each oocyte and each CC sample were sequenced at an average depth of approximately 0.7 million reads per oocyte and 2.0 million per CC sample (Supplementary Figure 1D), and the average ratio mapped of each oocyte and each CC sample was 11.55 and 16.58%, respectively (Supplementary Figure 1E). The DESeq2 was subsequently applied to identify differentially expressed TEs, and only the TEs with a fold change of log2 transformed larger than log_2_(1.5) and adjusted *P*-value < 0.05 from DEseq2 results were considered to be differentially expressed, and the expression of TEs was normalized by DESeq2. The normalized expression of TEs was used as input for t-SNE dimension reduction.

### Correlation Analysis

We screened genes with −log_10_ (padj) > 10 from the differential gene expression of oocytes, showing the most significantly upregulated protein-coding genes in PCOS oocytes, and then calculated the average normalized expression of these genes in each oocyte (*G_i_*). By counting the proportion of differentially expressed TEs on each chromosome according to the classification of the classes and super-families, we found out the super-families significantly enriched and upregulated in PCOS oocytes and then calculated the average normalized expression of the super-families in each oocyte (*T_i_*). Log2-transformed *G_i_* values and log2-transformed *T_i_* values were used as inputs for correlation analysis using Pearson’s correlation. The plot was drawn by the R package “pheatmap.”

### Oocyte *in vitro* Maturation

The *in vitro* maturation of GV oocytes was achieved using SAGE IVM media kit (ART-1600). Briefly, GV oocytes surrounded by CCs, retrieved during IVF cycles, were washed twice with SAGE washing medium and then cultured in SAGE IVM medium with 75 IU human menopausal gonadotropin for approximately 24 h under paraffin oil at 37°C in a highly humidified atmosphere of 6% CO_2_ in air (Fesahat et al., 2017). Oocyte maturation was assessed by the presence of the first polar body using a stereomicroscope. For immunofluorescence microscopy of oocytes, the surrounding CCs were removed by hyaluronic acid.

### Immunofluorescence and Confocal Microscopy

Immunofluorescence microscopy of oocytes for spindle imaging was performed based on the previous method (Allworth and Albertini, 1993). The oocytes were fixed in fixative (MTSB XF) at 37°C for at least 30 min and then washed four times with washing buffer (PBS, supplemented with 0.02% NaN3, 0.01% Triton X-100, 0.2% non-fat dry milk, 2% goat serum, 2% bovine serum albumin, and 0.1 M glycine). Afterward, the oocytes were left in the washing buffer for 2 h at 37°C for blocking. To determine protein expression, the oocytes were incubated with anti-TUBB8 antibody (1:500, SAB2700070, Sigma-Aldrich) or anti-TUBA1C (1:300, PA516891, Thermo Fisher Scientific) overnight at 4°C. The oocytes were washed and incubated with secondary goat anti-rabbit IgG Alexa Fluor 594 antibody (1:200, 111-585-003, Jackson) at 37°C for 2 h and Hoechst 33342 (1:200, H3570, Life Technologies) to label the DNA. The oocytes were mounted on glass slides, sealed with nail polish, and examined with a confocal laser scanning microscope (Leica). The oocytes not incubated with anti-TUBB8 or TUBA1C antibodies but only with the secondary antibody and stained DNA with Hoechst 33342 served as control for non-specific staining. The control oocytes showed only DNA staining (in blue) and no other non-specific staining.

### Gene Expression Analysis by Real-Time Quantitative PCR

The mRNA of CCs was reverse-transcribed to cDNA according to the Smart-seq2, and the products were diluted at a final concentration of 0.25 ng/μl. qPCR reactions were performed in duplicate with the FS Universal SYBR Green Master (4913914001, Roche) and run on the iCycler MyiQ2 Detection System (Bio-Rad). Each sample was repeated three times and analyzed using GAPDH as the internal control. The amplification program was set up as follows: primary denaturation at 95°C for 10 min, then 40 cycles of denaturation at 95°C for 15 s, annealing and elongation at 58°C for 1 min, and last cycle for dissociation curve under 55–95°C. The primers used for qPCR are listed in Supplementary Table 3, and their specificity was confirmed with a dissociation curve.

### Permutational Multivariate Analysis of Variance

Differences of global gene or TE expression between the PCOS and control groups were tested by permutational multivariate analysis of variance (PerMANOVA) as implemented by the function adonis in the R package vegan (v2.5-6) based on the Bray–Curtis distance measure (permutation:999).

### Statistical Analysis

Data for gene expression levels was analyzed by Student’s *t*-test (paired comparison) and ANOVA (multiple comparisons) using StatView software from SAS Institute Inc. (Cary, NC, United States). The results are represented as mean ± SD, and the *P* values for these statistical analyses were based on three oocytes in duplicate or two CC samples in duplicate from three PCOS patients or three controls. Data for clinical characteristics and hormone levels between PCOS patients and controls was analyzed by unpaired Student’s *t*-test using the SPSS 26.0 software and shown as mean ± SEM (*n* = 5). Significant differences were defined as ^∗^*P* < 0.05, ^∗∗^*P* < 0.01, or ^∗∗∗^*P* < 0.001.

## Results

### Global Gene Expression Profile of Oocytes Distinguishes PCOS From Control

The study included five PCOS patients and five controls, and the clinical characteristics are shown in Supplementary Table 1. The PCOS patients exhibited a significantly higher LH level (7.71 ± 1.11 mIU/ml) than controls (3.93 ± 0.65 mIU/ml). Compared to controls (FSH: 6.99 ± 0.56 mIU/ml, *T*: 28.80 ± 7.23 ng/ml, E2: 3,272.60 ± 603.52 pg/ml), the PCOS patients had lower FSH levels (4.91 ± 0.46 mIU/ml), elevated testosterone (52.87 ± 3.93 ng/ml), and higher E2 levels (5,813.53 ± 298.03 pg/ml). In addition, the number of antral follicles and oocytes retrieved from PCOS patients (31.40 ± 1.69 and 34 ± 7.62, respectively) was significantly more than that of controls (16.80 ± 3.40 and 15.20 ± 2.78, respectively). Next, we performed RNA sequencing of six GV oocytes and four CC samples from the same PCOS patients or controls. The sequencing depth of libraries for all oocytes was sufficient to ensure the accuracy and consistency of a subsequent analysis. After quality control and filtration of the RNA-seq data, we identified 24,251 genes expressed in oocytes and 42,725 genes in CC samples. The obtained RNA-seq normalized data were used for dimensional reduction analysis (t-SNE) (van der Maaten and Hinton, 2008) by an unsupervised approach, showing that there was a clear separation between oocytes and matched CCs with or without PCOS (Supplementary Figure 1F). Interestingly, we found distinct PCOS-specific clustering of oocytes, whereas the clustering of CCs displayed some overlap. Meanwhile, PerMANOVA further confirmed significant differences in the global gene expression of oocytes or CCs between the PCOS and control groups (Supplementary Table 4, *P* = 0.004, *P* = 0.03). Hence, oocytes and CCs had different gene expression patterns in the occurrence of PCOS.

**FIGURE 1:**
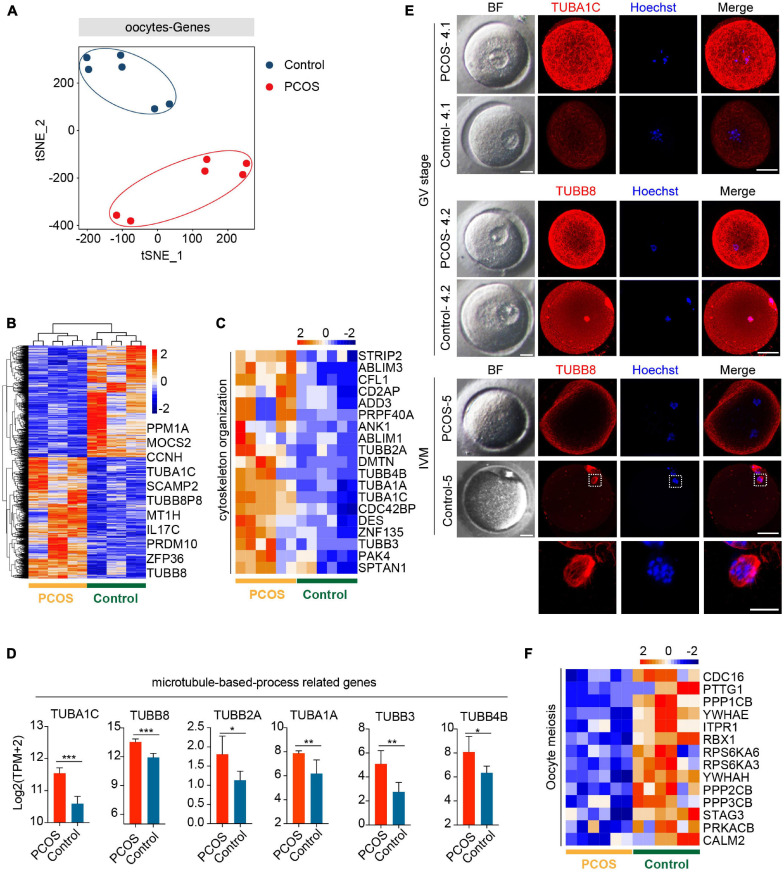
Global gene expression profile distinguishes polycystic ovary syndrome (PCOS) from control oocytes. **(A)** Visualization of the gene expression of six oocytes by t-SNE, which are clustered into two subpopulations including the control group and the PCOS group; the red and blue points represent PCOS and control oocytes, respectively. **(B)** Heat map displaying differentially expressed genes (DEGs) in oocytes between PCOS and control. The number of up-DEGs is 1,433 and down-DEGs is 1,322. The color key from blue to red indicates the relative gene expression levels from low to high, respectively. **(C)** Heat map of genes involved in cytoskeleton organization that were upregulated in PCOS oocytes. **(D)** Six genes associated with a microtubule-based process. The red and blue bars represent PCOS and control, respectively. The gene expression levels are represented by log_2_ [TPM + 2], and data represents mean ± SD. *n* = 3 (participants). *p < 0.05, **p < 0.01, ***p < 0.001, ns, not significant. **(E)** Bright-field and immunofluorescence images of TUBA1C and TUBB8 in normal and PCOS oocytes at the germinal vesicle (GV) stage or metaphase II (MII) stage after *in vitro* maturation. Upper panel, oocytes (PCOS and control) at GV stage and two PCOS oocytes or two control oocytes were obtained from the same person. Lower panel, oocytes (PCOS and control) after *in vitro* maturation. Hoechst (blue) was used to counterstain and visualize DNA. Scale bar represents 20 μm. The magnifications of the spindle regions are shown at the bottom, and the scale bar is 10 μm (bottom). **(F)** Heat map of genes associated with oocyte meiosis downregulated in PCOS oocytes.

To explore the pattern of gene expression in oocytes from PCOS, we applied t-SNE to the normalized expression data by using unsupervised clustering. The PCOS and control clusters could be noticeably differentiated (Figure 1A). Next, we examined DEGs between PCOS (1,433 genes upregulated and 1,322 genes downregulated) and control oocytes (Figure 1B and Supplementary Table 5). Although these data were only generated from three PCOS patients and three controls, such consistent gene expression patterns among them shown in the heatmap demonstrated the robustness of our RNA-seq analysis as well as minimal variations among patients. By GO analysis, crucial functions (*P* < 0.05) enriched by upregulated DEGs are displayed (Supplementary Figure 1G). It is noteworthy that genes associated with the function of chromatin, microtubule, cytoskeleton, and actin were upregulated in oocytes from PCOS women (Figures 1C,D). Among genes involved in the microtubule-based process, *TUBB8* and *TUBA1C* exhibited the highest expression levels in PCOS oocytes. The immunofluorescence of oocytes confirmed TUBB8 and TUBA1C overexpression in oocytes from women with PCOS (Figure 1E). It has been found that TUBB8 plays a key role in meiotic spindle assembly and maturation in human oocytes, and mutations in TUBB8 lead to oocyte maturation arrest (Feng et al., 2016; Chen et al., 2017).

Meanwhile, the pivotal function (*P* < 0.05) enriched by downregulated DEGs was also revealed (Supplementary Figure 1H). By KEGG analysis, significantly downregulated signaling pathways in PCOS oocytes included the MAPK, mTOR, and FOXO signaling pathways (Supplementary Figure 1I). The MAPK signaling pathway is important for the cell cycle of human oocytes (Pal et al., 1994; Sun et al., 1999), and the mTOR–eIF4F pathway spatiotemporally regulates the translation of mammalian oocytes in meiosis (Susor et al., 2015). Upregulated genes were also enriched for the signaling pathways (*P* < 0.05) of spliceosome and gap junctions (Supplementary Figure 1J). Gap junctions play an important role in the communication between oocytes and CCs, and the breakdown of the gap junction in the ovarian follicle induces a recommencement of meiosis (Sela-Abramovich et al., 2006; Gershon et al., 2008).

Based on the above-mentioned GO and KEGG analysis of oocytes from PCOS, we considered that a disordered expression of several signaling pathways and a dysfunction of the cytoskeleton, specifically microtubules, may result in meiotic abnormality in the oocytes from patients with PCOS. Indeed while control oocytes reached MII with clearly visible, barrel-shaped spindle with well-aligned chromosomes, PCOS oocytes displayed maturation arrest and no spindles as well as disrupted TUBB8 (Figure 1E). Genes involved in meiosis were also downregulated in PCOS oocytes (Figure 1F). Taken together, our data revealed that the aberrant gene expression profile, including cytoskeleton, microtubule, and meiosis, likely impairs the developmental competence of oocytes from patients with PCOS.

### Gene Expression Pattern of CCs During the Occurrence of PCOS

**FIGURE 2:**
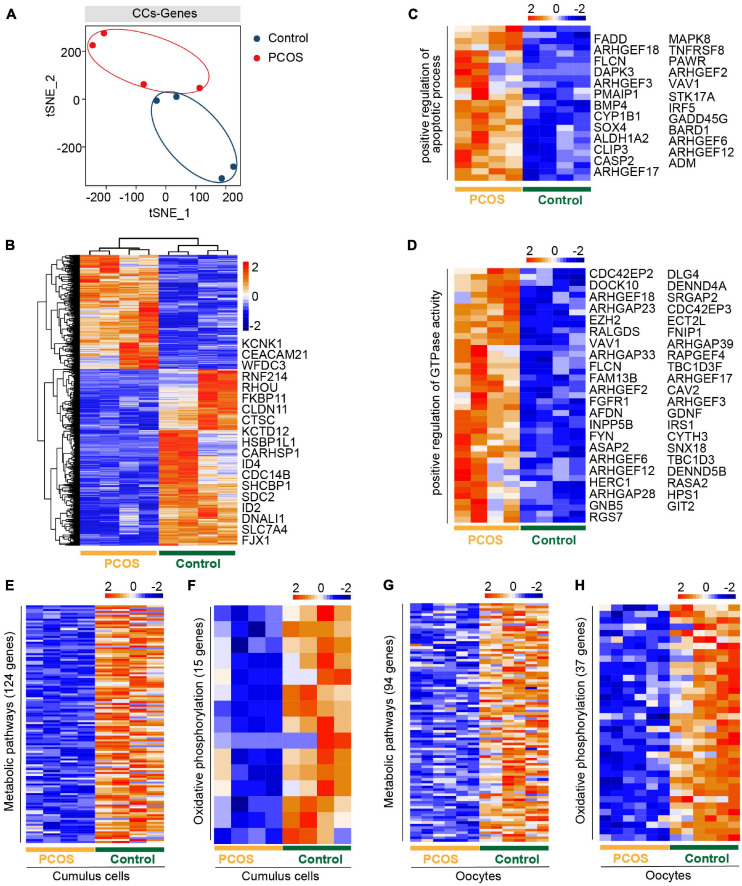
The gene expression pattern differs between PCOS and control cumulus cells (CCs). **(A)** Visualization of gene expression by RNA-seq from four CC samples by t-SNE, showing that CCs are clustered into two distinct subpopulations. The red and blue points represent PCOS and control CCs, respectively. **(B)** Heat map of DEGs in CCs between PCOS and control. The number of up-DEGs is 704 and down-DEGs is 1,091. The color key from blue to red indicates the relative gene expression levels from low to high, respectively. **(C)** Heatmap of genes involved in the positive regulation of apoptotic process upregulated in PCOS CCs. **(D)** Heat map of genes related to the positive regulation of GTPase activity upregulated in PCOS CCs. **(E)** Heat map of genes involved in metabolic pathways downregulated in PCOS CCs. **(F)** Heat map of genes involved in oxidative phosphorylation downregulated in PCOS CCs. **(G)** Heat map of genes related to metabolic pathways downregulated in PCOS oocytes. **(H)** Heat map of genes related to oxidative phosphorylation downregulated in PCOS oocytes.

An intimate communication between oocytes and CCs plays a crucial role in follicle development and oogenesis (Canipari, 2000). We also performed an RNA-seq analysis of CCs from the same patients who donated the oocytes. All CCs were clustered into two groups, depending on whether suffering from PCOS or not (Figure 2A). The clear separation of the t-SNE pattern indicated a pronounced difference in the transcriptome of PCOS and control CCs. We then characterized DEGs between PCOS CCs (704 upregulated genes and 1,091 downregulated genes) and control CCs (Figure 2B and Supplementary Table 6). The GO terms (biological process, BP) enriched by upregulated DEGs were found in PCOS CCs (Supplementary Figure 2A). Several genes associated with the positive regulation of the apoptotic process demonstrated an increased expression level in PCOS CCs (Figure 2C). Genes associated with a positive regulation of GTPase activity were also overexpressed in CCs from women with PCOS (Figure 2D).

Moreover, the downregulated DEGs included oxidative reduction and glycogen biosynthetic processes and response to hypoxia (Supplementary Figure 2B). The decreased expression levels of “response to estrogen”-related genes may explain why patients with PCOS are less sensitive to estrogen and have increased T levels (Supplementary Table 1), supporting the increased androgen biosynthesis in PCOS theca cells and the inhibition of aromatase in granulosa cells (Nelson et al., 1999; Magoffin, 2006).

By KEGG analysis of crucial signaling pathways, strikingly, among these signaling pathways (Supplementary Figures 2C,D), many genes involved in metabolic and oxidative phosphorylation pathways showed a decreased expression in CCs from women with PCOS (Figures 2E,F), consistent with those of oocytes from PCOS women (Figures 2G,H). However, the genes of metabolic pathways and oxidative phosphorylation were not the same in CCs and oocytes, owing to their different cell types. Some genes involved in the PI3K-Akt signaling pathway displayed high expression levels in CCs from PCOS patients (Supplementary Figure 2E). In addition, genes related to the MAPK and Ras signaling pathways were also expressed at increased levels in PCOS CCs (Supplementary Figures 2F,G). Our analysis revealed a simultaneous dysfunction of oxidative phosphorylation and metabolic pathways in both CCs and oocytes, in addition to the changes in previously known signaling pathways found in granulosa cells or CCs.

### Disorder of Mitochondrial Function and Communication in PCOS Oocytes and CCs

The bidirectional communication between oocytes and their associated somatic cells plays a pivotal role in fertility and embryogenesis (Matzuk et al., 2002). To investigate whether the crosstalk is dysfunctional between oocytes and CCs in PCOS, we explored the signaling pathways that are potentially involved in the interaction between oocytes and CCs. The NOTCH signaling pathway, such as ligands DLL3 and JAG2, had no significant difference in oocytes between PCOS and controls, and only receptor NOTCH3 and target gene HES1 were differentially expressed in PCOS CCs (Supplementary Figure 3A). In terms of the gap junction, the expression of three key genes related to this pathway, including GJC1, GJA3, and GJA1, did not differ in CCs with PCOS and controls (Supplementary Figure 3B). The ligand KITLG and the receptor KIT displayed increased expression levels in CCs and oocytes from PCOS patients, respectively (Supplementary Figure 3C).

**FIGURE 3:**
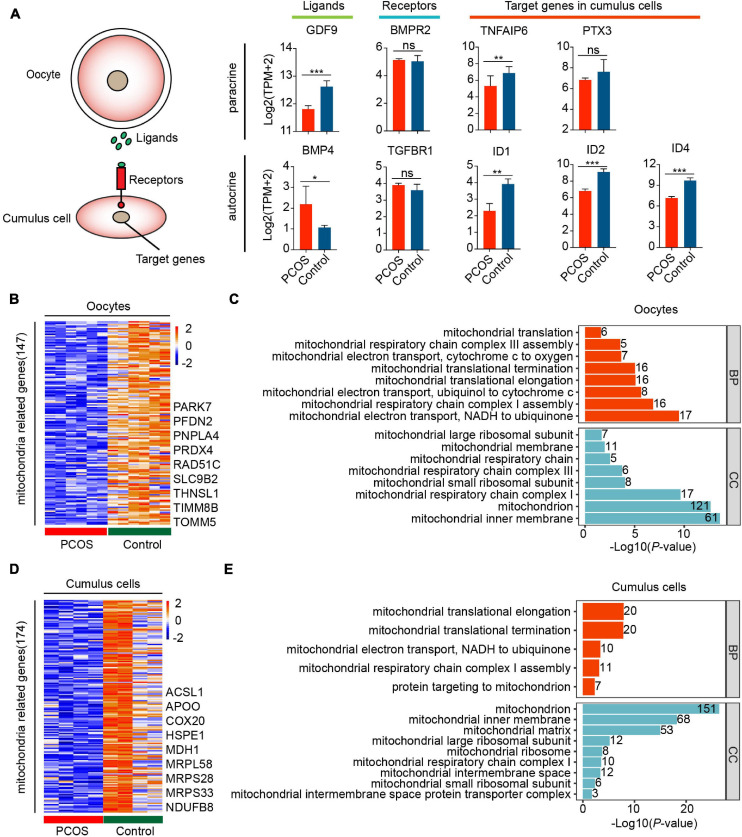
Disorder of mitochondrial function and communication in CCs and oocytes with PCOS. **(A)** The TGF-β signaling pathway is involved in the oocyte–GC interactions. The schematic diagram at the left shows the relationship among these genes. The diagrams at the right show the relative expression levels [log2 ([TPM+2)] of ligands, receptors, and target genes between PCOS and control in oocytes (*n* = 3 participants) and CCs (*n* = 2 participants). Data represents mean ± SD. *p < 0.05, **p < 0.01, ***p < 0.001, ns, not significant. **(B)** Heat map of DEGs involved in the mitochondria that were downregulated in oocytes with PCOS. **(C)** Gene ontology (GO) enrichment analysis of downregulated mitochondria genes in PCOS oocytes. The red bars represent biological process (BP), and the blue bars represent cellular component (CC). **(D)** Heat map of DEGs involved in mitochondria downregulated in PCOS CCs. **(E)** GO enrichment analysis of downregulated mitochondria genes in PCOS CCs.

Remarkably, the ligand GDF9 in oocytes was downregulated in the TGF-β signaling pathway, whereas the ligand BMP4 was upregulated in the CCs of PCOS patients. Although the expression of receptors did not differ in CCs, the target genes correlating with cumulus–oophorus extracellular matrix and luteal function, including TNFAIP6, PTX3, ID1, ID2, and ID4, were downregulated in PCOS CCs (Figure 3A). This suggests incomplete cumulus expansion and aberrant luteinization, which presumably affects ovulation in women with PCOS. Overall, the bidirectional communication between the oocytes and CCs may be disrupted.

The above-mentioned functional enrichment analysis of oocytes and CCs demonstrated alterations in genes related to metabolism and oxidative phosphorylation. Furthermore, 147 of genes involved in mitochondrial function were downregulated in PCOS oocytes (Figure 3B and Supplementary Table 7). A total of 174 genes associated with mitochondrial function were also downregulated in PCOS CCs (Figure 3D and Supplementary Table 8), and the CCs and oocytes shared 18 genes related to the mitochondria. The downregulation of mitochondria-related genes such as NDRG4, UCP2, and MRPS26 by RNA-seq was further validated by real-time qPCR (Supplementary Figure 3D). We also investigated mitochondrial function and components by GO analysis, including BP and cellular component. The downregulated mitochondrial genes in oocytes and CCs were enriched for the same cellular component terms, including mitochondrial inner membrane, mitochondrial large ribosomal subunit, mitochondrial respiratory chain complex I, and mitochondrial small ribosomal subunit (Figures 3C,E). Furthermore, several BP terms were also enriched in oocytes and CCs, including mitochondrial translational elongation/termination, mitochondrial respiratory chain complex I assembly and mitochondrial electron transport, and NADH to ubiquinone. Globally, the GO analysis of mitochondria-related genes highlighted that mitochondrial dysfunction and faulty mitochondrial components simultaneously occur in oocytes and CCs in women with PCOS, which might contribute to oocytes with decreased fertilization and impaired embryonic development.

### Expression Pattern of TEs in Oocytes Between PCOS and Control

**FIGURE 4:**
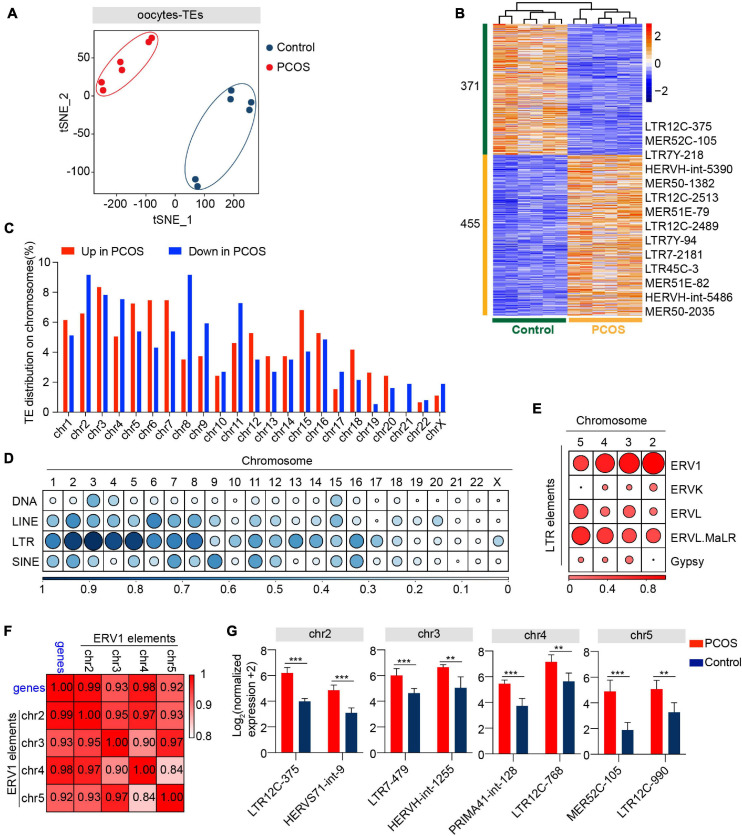
Retrotransposons in PCOS and control oocytes. **(A)** Visualization of transposable element (TE) expression of oocytes by t-SNE, showing two clusters, including one for PCOS oocytes and the other for control oocytes (*n* = 6). Each dot corresponds to one oocyte, clustered by color. **(B)** Heat map of differentially expressed TEs (DETs) in oocytes between PCOS and control. The number of up-DETs is 455 and down-DETs is 371. The color key from blue to red indicates the relative gene expression levels from low to high, respectively. **(C)** The proportion of differentially expressed TEs in each chromosome. The red bars represent the percentage of upregulated TEs, and the blue bars represent the percentage of downregulated TEs. **(D)** Proportion of DETs classified by repeat classes on different chromosomes. Dots from large to small or color from dark to light represents the proportion from high to low. **(E)** Proportion of differentially expressed long terminal repeat (LTR) elements classified by repeat super-families on chromosomes 2, 3, 4, and 5. **(F)** Correlation between the expression levels of ERV1 elements on chromosomes 2, 3, 4, and 5 and of protein-coding genes in PCOS oocytes. The correlation coefficient is shown on the plot. **(G)** The expression levels of differentially expressed ERV1 elements on chromosomes 2, 3, 4, and 5 in PCOS oocytes, and also shown are two differentially expressed ERV1 elements on each chromosome.

In addition to alteration of gene expression in oocytes and CCs, we also investigated whether TEs are involved in the pathogenesis of PCOS. Two clusters representing PCOS or control were clearly identified (Figure 4A), and PerMANOVA also demonstrated that the expression pattern of TEs can distinguish PCOS from control oocytes (Supplementary Table 4, *P* = 0.004). The PCOS oocytes showed 455 upregulated and 371 downregulated TEs (Figure 4B and Supplementary Table 9). Most upregulated or downregulated TEs in PCOS oocytes belonged to LTR elements, LINEs, and SINEs (Supplementary Figure 4A). The LTR elements accounted for the largest proportion of main classes. The proportion of downregulated TEs (43.08%) classified in LTR elements was slightly higher than the upregulated TEs (39.08%). In terms of superfamily, the differentially expressed TEs in PCOS oocytes mainly pertained to ERV1, ERVL-MaLR, Alu, L1, and hAT-Charlie. The upregulated TEs belonged to ERV1 and ERVL-MaLR, in contrast to L1, Alu, and hAT-Charlie (Supplementary Figure 4B). We further analyzed LTR retrotransposons which are proportionately greater than other repeat classes. Approximately 24% of ERV1 elements were highly expressed in PCOS oocytes, including LTR1C, LTR16C, MLT1J2, MER4C, and MLT1G1, in contrast to the 11% downregulated expression of ERV1 elements, including LTR12C, MLT1H1, MER41B, and LTR7Y (Supplementary Figure 4C).

To analyze the chromosomal distribution of differentially expressed TEs, we classified differentially expressed TEs according to the chromosome position (chromosome 1-22, X chromosome) (Figure 4C). The differentially expressed TEs were not uniformly dispersed in the human chromosomes. Most differentially expressed TEs were distributed on chromosomes 1–8. In contrast, TEs on chromosomes 20, 21, and 22 and X chromosome were less differentially regulated (Supplementary Figure 4D). Moreover, differentially expressed TEs were mainly enriched in LTR elements on chromosomes 2, 3, 4, and 5 (Figure 4D). The LTR elements were composed of Gypsy, Copia, Bel-Pao, retrovirus, and ERV, based on the classification system for TEs (Wicker et al., 2007). Notably, most differentially expressed LTR elements on chromosomes 2, 3, 4, and 5 were classified as ERV1 elements (Figure 4E). To confirm whether these differentially expressed ERV1 elements were involved in the occurrence of PCOS, we screened 13 most significantly upregulated genes with −log_10_ (padj) > 10 from the DEGs between PCOS oocytes and controls (Supplementary Table 10) and then performed a correlation analysis by Pearson’s correlation between the average normalized expression levels of 13 most significant PCOS-specific protein-coding genes and of the upregulated ERV1 elements on chromosomes 2, 3, 4, and 5 in PCOS oocytes. Strikingly, the highly upregulated ERV1 elements on chromosomes 2, 3, 4, and 5 are significantly correlated with those of 13 protein-coding genes notably including tubulin-associated genes *TUBA1C*, *TUBB8P8*, and *TUBB8* (Figure 4F) and these ERV1 elements were activated in PCOS oocytes (Figure 4G), suggesting that ERV1 elements may be involved in the alterations in gene regulation observed in PCOS.

### TE Expression Profile in CCs Between PCOS and Control

**FIGURE 5:**
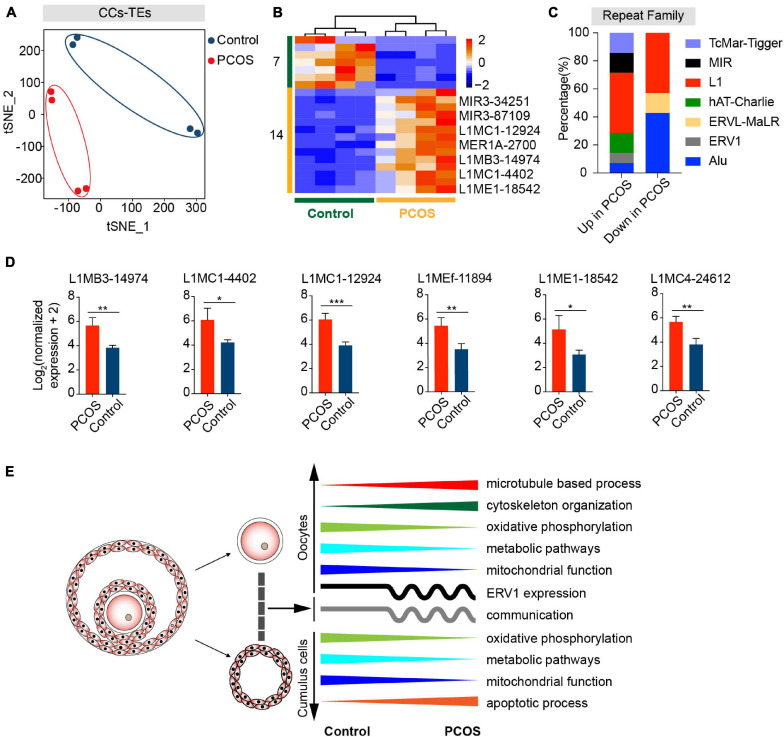
Retrotransposons in PCOS and control CCs. **(A)** Visualization of TE expression of CCs by t-SNE, showing two clusters, including one for PCOS CCs (four samples) and the other for control CCs (four samples). Each dot corresponds to CCs. **(B)** Heat map of all differentially expressed TEs (DETs) in CCs between PCOS and control. The number of up-DETs is 14 and down-DETs is 7. **(C)** Percentage of upregulated or downregulated TEs classified by repeat superfamily. **(D)** Expression levels of differentially expressed L1 elements in PCOS CCs. **(E)** Summary of molecular features in oocytes and CCs in the occurrence of PCOS.

We wondered whether TEs are also differentially expressed in PCOS CCs. By t-SNE clusters, the transcription of TEs in PCOS CCs clearly was distinguishable from that of the control (Figure 5A). The overall alteration of TE expression profile in PCOS CCs was not as dramatic as in PCOS oocytes, and the TE expression profile in PCOS CCs did not differ from that of controls by PerMANOVA (Supplementary Table 4, *P* = 0.280). Compared with control CCs, 14 differentially expressed TEs displayed upregulation and seven displayed downregulation in PCOS CCs (Figure 5B and Supplementary Table 11). Most differentially expressed TEs were enriched in L1 elements (Supplementary Figure 4E and Figure 5C). L1 retrotransposons are autonomously active and can integrate into the genome, which can remodel the gene structure and further impact human evolution and disease (Sultana et al., 2019). Most differentially expressed L1 elements exhibited high expression levels in PCOS CCs, containing L1MC1, L1MB3, L1MEf, L1ME1, and L1MC4 (Figure 5D). The role of CC TEs in PCOS remains to be determined, though we cannot rule out the possibility that TEs may influence the transcription of genes implicated in PCOS.

## Discussion

By simultaneous transcriptome analysis of the oocytes and CCs from the same PCOS patient, compared with controls at similar age, we identified differences in global gene expression patterns and in the expression of TEs in oocytes or CCs in PCOS patients (Figure 5E). Our data revealed surprising findings of altered microtubule-related genes in addition to altered metabolisms, as reported previously. We also discovered changes in the transcription of retrotransposons, which would be likely to influence the transcriptome of PCOS oocytes. ERV1 elements particularly may be involved in the pathogenesis of PCOS. In contrast, CCs from PCOS patients contained fewer alterations in TEs. This data also supports the notion that most retrotransposon events occur in parental germ cells and only rarely in somatic cells (Kazazian, 2004; Beck et al., 2010).

Previous studies on the transcriptomic profiles of oocytes and granulosa cells or CCs from PCOS patients have shown molecular abnormalities present in PCOS patients. Wood et al. (2007) reported that several DEGs of PCOS oocytes are related to spindle dynamics and centrosome function. A similar study using single-cell RNA-seq found that some genes involved in meiosis and gap junction exhibited an abnormal expression in PCOS oocytes (Liu et al., 2016). Here we find that genes involved in microtubule-based processes, e.g., cytoskeleton organization, were differentially expressed in PCOS oocytes. Specifically, crucial genes involved in microtubule-based processes, *TUBB8* and *TUBA1C*, are overexpressed in PCOS oocytes. These changes would be expected to negatively affect oocyte maturation and the subsequent embryonic development. Interestingly, mutations in *TUBB8* contribute to the disruption of oocyte meiotic spindle assembly and maturation and lead to female infertility (Feng et al., 2016). Subsequent studies have also proved through a mutation analysis that the mutation of *TUBB8* is related to female infertility, and the mutation causes abnormal phenotypes, including oocyte maturation arrest, oocytes with large polar body, failure of fertilization, and early embryonic arrest (Yuan et al., 2018; Chen et al., 2019; Jia et al., 2020; Zhao et al., 2020). Although PCOS patients have more oocyte yield from stimulation during IVF cycle, poor oocyte quality causes lower fertilization and implantation rates, poor quality of embryos, decreased pregnancy rates, and increased miscarriage rates (Dor et al., 1990; Sengoku et al., 1997; Mulders et al., 2003; Plachot et al., 2003; Heijnen et al., 2006; Weghofer et al., 2007; Sahu et al., 2008; Qiao and Feng, 2011; Rajani et al., 2012). Indeed oocytes from women with PCOS displayed maturation arrest and disrupted spindles after *in vitro* maturation, as shown in our study. Hence, the increased expression of *TUBB8* and *TUBA1C* resulting in defective microtubule and spindle formation could be one major factor contributing to the decline in oocyte quality in PCOS women.

In addition, crucial signaling pathways are altered in oocytes from PCOS women – for instance, genes related to the MAPK signaling pathway show a decreased expression in oocytes from women with PCOS, and MAPK plays a pivotal role in regulating oocyte meiotic resumption (Edry et al., 2006; Liang et al., 2007). The expression of genes involved in mTOR signaling is also decreased in oocytes from PCOS women. The mTOR–eIF4F pathway spatiotemporally regulates chromosome segregation and functional spindle formation during meiosis in mammalian oocytes (Susor et al., 2015). However, it is unclear whether aberrant MAPK and mTOR are related to the overexpression of microtubule-associated genes in PCOS.

Our findings also imply the potential involvement of TEs in PCOS oocytes and CCs. The pattern of TE expression distinguishes PCOS from control oocytes. Most differentially expressed TEs are classified as LTR elements belonging to the super-families ERV1, ERVL-MaLR, Alu, L1, and hAT-Charlie. ERV1 elements differentially expressed on chromosomes 2, 3, 4, and 5 in oocytes may also be associated with PCOS. Remarkably, ERVs are highly expressed in models of reproductive defects – for instance, *Setdb1* deletion early in germline development leads to gametogenesis defects in postnatal and adult mice, and ERV is reactivated in E13.5 primordial germ cells (Liu et al., 2014). ERV is upregulated in *Tex19.1* knockout mice which have defects in meiotic chromosome synapsis (Ollinger et al., 2008). Future studies should determine whether ERV1 can serve as a biomarker of PCOS. In addition, fewer TEs are altered in CCs than in oocytes. It will be interesting to determine whether these L1s contribute to genome instability in women with PCOS.

Most of previous studies on PCOS focused on ovarian somatic cells, peripheral blood, or other cell types – for instance, CCs of PCOS patients display abnormal characteristics of gene expression, including dysregulated growth factors, steroid metabolism, cell cycle, steroid hormone biosynthesis, and hypomethylated genes related to the synthesis of lipid and steroid (Haouzi et al., 2012; Wissing et al., 2014; Liu et al., 2016; Pan et al., 2018). Dysregulation of inflammatory function has also been found in PCOS patients through transcriptome and DNA methylation analysis of peripheral blood and granulosa-lutein cells (Adams et al., 2016; Su et al., 2018; Hiam et al., 2019). In addition, the aberrant expression of MicroRNAs in granulosa cells, theca cells, and follicular fluid might be involved in the development of PCOS (Roth et al., 2014; Jiang et al., 2015; Lin et al., 2015). In our study, global gene expression in CCs from PCOS women also differs from controls. Furthermore, two key genes are differentially expressed in CCs from PCOS patients, including LH/choriogonadotropin receptor and insulin receptor gene (Supplementary Table 6), which were identified as susceptibility genes for PCOS in previous GWAS and strongly associated with anovulation (Chen et al., 2011; Shi et al., 2012; Cui et al., 2015). The functional annotation of genes upregulated in PCOS CCs shows alteration in the positive regulation of GTPase activity, apoptotic process, and steroid metabolic process. Alteration of gene expression related to “positive regulation of apoptotic process” may activate the apoptosis of CCs and indirectly reduce the quality and developmental competence of the oocytes (Corn et al., 2005). Conversely, genes related to the carbohydrate metabolic process and response to lipopolysaccharide show a decreased expression. The abnormal metabolism in CCs is thought to contribute to the clinical features shared among PCOS, obesity, and diabetes. In support, PCOS women have increased prevalence of metabolic syndrome, impaired glucose tolerance, and obesity (Ehrmann, 2005; Moran et al., 2010; Lim et al., 2012; Ho et al., 2019). The ovarian reserve may also be affected by obesity in women variably depending on the presence of PCOS (Kim et al., 2020). Meanwhile, some signaling pathways were also dysregulated in CCs from PCOS patients, such as the PI3K-Akt and MAPK signaling pathways, which are known to be related to PCOS, involving insulin resistance and excessive androgen production (Nelson-Degrave et al., 2005; Hojlund et al., 2008; Aydos et al., 2016; Li et al., 2017).

Our finding reveals that metabolic pathways and oxidative phosphorylation are dysregulated in both oocytes and CCs of PCOS patients, further validating and explaining the phenotype that PCOS women exhibit an increased risk of metabolic syndrome. Anovulation is a common cause of infertility in PCOS patients, and some studies have shown that anovulatory infertility is related to metabolic abnormalities (Clark et al., 1995; Cardozo et al., 2011; Balen et al., 2016). Thus, for women with anovulatory PCOS, abnormal metabolism in CCs may be related to anovulation. Moreover, disordered mitochondrial function in oocytes and CCs may contribute to declined oocyte developmental competence. The mitochondria are essential for oocyte development potential and oocyte rejuvenation (Labarta et al., 2019), and mitochondrial dysfunction in oocytes is found in women with PCOS (Ou et al., 2012; Zhang et al., 2019). Mitochondrial functions may be prematurely activated at GV-stage oocytes of PCOS (Qi et al., 2020), and the oocytes exhibit impaired mitochondrial ultrastructure and functions, including compromised inner mitochondrial membrane potential and electron transport chain (Chappell et al., 2020), consistent with our results. The crosstalk between oocytes and CCs can also be perturbed by the alteration of the ovarian microenvironment, including oxidative stress caused by mitochondrial respiratory dysfunction, which leads to enhanced ROS production (May-Panloup et al., 2016). Additionally, our analysis reveals that the communication between oocytes and CCs may be disrupted in PCOS patients, notably members of the TGF-β superfamily, including BMPs/GDFs, that are important regulators in human folliculogenesis and ovulation (Chang et al., 2016). It is likely that the abnormalities of essential signaling pathways may attribute to the disorder of CCs and further influence the quality of oocytes in PCOS patients.

Our study comprehensively elaborates the molecular features of PCOS by the transcriptomic analysis of oocytes and CCs and their interactions from the same patient. Although our sample size is small, the clinical characteristics of women with PCOS are consistent with those reported in previous articles (Qiao and Feng, 2011; Liu et al., 2016), and clinical data-related tests including normality and variance equality also further prove the consistency of our data (Supplementary Figures 5A,B). Moreover, the RNA-seq data is highly consistent among patients, demonstrating the reliability of the technology and the minimal variations in their gene expression profile. To our knowledge, this study is the first interpretation of the relationship between PCOS and retrotransposons in oocytes. Furthermore, new candidate genes and TEs in oocytes and CCs may serve as the signatures of PCOS. Increased expression levels of *TUBB8* and *TUBA1C* and resultant spindle defects can specifically define the oocyte quality of PCOS patients. Overall, these findings may suggest future treatment strategies to improve oocyte maturation and developmental competence in PCOS women. The underlying mechanisms of aberrantly elevated *TUBB8* and *TUBA1C* and also *ERV1* in PCOS remain to be understood.

## Data Availability Statement

The datasets presented in this study can be found in online repositories. The names of the repository/repositories and accession number(s) can be found below: GEO: GSE155489.

## Ethics Statement

The studies involving human participants were reviewed and approved by the Ethics Committee of the Tianjin Medical University General Hospital. The patients/participants provided their written informed consent to participate in this study.

## Author Contributions

JL and HC conducted the experiments. JL prepared the manuscript. MG, CT, and HW conducted part of the experiments or provided reagents. XS, DK, and XB designed and discussed the experiments and revised the manuscript. LL conceived the project and revised the manuscript. All authors contributed to the article and approved the submitted version.

## Conflict of Interest

The authors declare that the research was conducted in the absence of any commercial or financial relationships that could be construed as a potential conflict of interest.

## Publisher’s Note

All claims expressed in this article are solely those of the authors and do not necessarily represent those of their affiliated organizations, or those of the publisher, the editors and the reviewers. Any product that may be evaluated in this article, or claim that may be made by its manufacturer, is not guaranteed or endorsed by the publisher.
