# Supplementary material for: Molecular Features of Polycystic Ovary Syndrome Revealed by Transcriptome Analysis of Oocytes and Cumulus Cells
Source: Front Cell Dev Biol. 2021 Sep 6;9:735684. doi: 10.3389/fcell.2021.735684 (PMC8450412; doi:10.3389/fcell.2021.735684)
Supplement: Supplementary file 2 [file Data_Sheet_2.docx]

**Supplementary data**

**Molecular features of polycystic ovary syndrome revealed by transcriptome analysis of oocytes and cumuluscells**

Jie Li et al.

**Supplementary Files:**

**Supplemental Table 1: Clinical characteristics and hormone levels of the participants.**

|  | **PCOS (n=5)** | **Control (n=5)** | ***P* value** |
| --- | --- | --- | --- |
| Age (years) | 32.40 ± 1.29 | 35.60 ± 2.23 | 0.249 |
| BMI (kg/m2) | 23.94 ± 1.46 | 20.76 ± 1.04 | 0.114 |
| LH (mIU/ml) | 7.71 ± 1.11 | 3.93 ± 0.65 | 0.018 |
| FSH (mIU/ml) | 4.91 ± 0.46 | 6.99 ± 0.56 | 0.021 |
| T (ng/ml) | 52.87± 3.93 | 28.80 ± 7.23 | 0.019 |
| AFC (n) | 31.40 ± 1.69 | 16.80 ± 3.40 | 0.005 |
| Starting rhFSH dose (IU) | 972.50 ± 107.41 | 1095 ± 152.97 | 0.531 |
| rhFSH stimulation duration (day) | 8.80 ± 1.32 | 10.00 ± 0.32 | 0.402 |
| Total rhFSH administrated (IU) | 1955 ± 367.71 | 2655 ± 505.68 | 0.295 |
| E2 level on hCG day (pg/ml) | 5813.53 ± 298.03 | 3272.60 ± 603.52 | 0.01 |
| Oocytes retrieved (n) | 34 ± 7.62 | 15.20 ± 2.78 | 0.049 |
| Follicles size (mm) | 15.44 ± 0.12 | 16.32 ± 0.62 | 0.233 |
| BMI (body mass index), LH (luteinizing hormone), FSH (follicle stimulating hormone), T (testosterone), AFC (antral follicle count), rhFSH (recombinant human follicle stimulating hormone). Data shown as the mean ± SEM; P-values determined by unpaired Student’s t-test. | | | |
|  |  |  |  |
|  |  |  |  |
|  |  |  |  |

**Supplemental Table 2: Samples for experiments.**

| **Sample** | **Oocyte(GV)** | **CCs** | **Experiment** |
| --- | --- | --- | --- |
| PCOS-1 | 1 | 1 | RNA-seq |
| PCOS-2 | 1 | 1 | RNA-seq |
| PCOS-3 | 1 | 0 | RNA-seq |
| PCOS-4 | 2 | 0 | GV-IF |
| PCOS-5 | 1 | 0 | GV-IVM+IF |
| Control-1 | 1 | 1 | RNA-seq |
| Control-2 | 1 | 1 | RNA-seq |
| Control-3 | 1 | 0 | RNA-seq |
| Control-4 | 2 | 0 | GV-IF |
| Control-5 | 1 | 0 | GV-IVM+IF |

IF (immunofluorescence), IVM (*in vitro* maturation)

**Supplemental Table 3: Primers for real-time qPCR.**

| **primer** | **sequence** |
| --- | --- |
| *UCP2*-F | AACAGTTCTACACCAAGGGC |
| *UCP2*-R | TCTTGTAGGCATTGACGGTG |
| *MRPS26*-F | CCAAATCCAAGATCGAGCGA |
| *MRPS26*-R | GACACGAACTCCATCCTGAG |
| *NDRG4*-F | GCATTTCGGGTTCAAGTATGTG |
| *NDRG4*-R | GTCGGGTAAAGTGCTAGTTAGG |
| *GAPDH*-F | ACAACTTTGGTATCGTGGAAGG |
| *GAPDH*-R | GCCATCACGCCACAGTTTC |

**Supplemental Table 4: PerMANOVA analysis by Bray Curtis distance measure.**

| Group | F model | R2 | P value |
| --- | --- | --- | --- |
| Genes of oocytes: PCOS / Control | 6.322880 | 0.387363 | 0.004 |
| TEs of oocytes: PCOS / Control | 3.449325 | 0.256468 | 0.004 |
| Genes of CCs: PCOS / Control | 2.627880 | 0.304580 | 0.030 |
| TEs of CCs: PCOS / Control | 1.167149 | 0.162847 | 0.280 |

**Supplementary Tables in excel sheet**

**Supplemental Table 5: Differentially expressed genes of oocytes between PCOS and Control.** Differentially expressed genes of oocytes between PCOS (N=6) and Control (N=6) are included in separate columns.

**Supplemental Table 6: Differentially expressed genes of CCs between PCOS and Control.** Differentially expressed genes of CCs between PCOS (N=4) and Control (N=4) are included in separate columns.

**Supplemental Table 7: Mitochondria-related genes differentially expressed in oocytes between PCOS and Control.**

**Supplemental Table 8: Mitochondria-related genes differentially expressed in CCs between PCOS and Control.**

**Supplemental Table 9: Differentially expressed transposable elements in oocytes between PCOS** (N=6) **and Control** (N=6)**.**

**Supplemental Table 10: 13 most significantly upregulated genes out of the differentially expressed genes between PCOS and Control oocytes.**

**Supplemental Table 11: Differentially expressed transposable elements of CCs between PCOS** (N=4) **and Control** (N=4)**.**

**Supplementary Figures**

**Figure S1:Quality control of RNA-seq analysis andkey enrichment analysis of oocytes in patients with PCOS**

**A,** Ct values of GAPDH from oocytes and CCs samples. **B,** Total mapped reads of genes from oocytes and CCs sample. **C,** Mapped ratio of genes from oocytes and CCs sample. **D,** Total mapped reads of TEs from oocytes and CCs sample. **E,** Mapped ratio of TEs from oocytes and CCs sample. **F**, Visualization of gene expression of six oocytes and four CCs samples by t-SNE, clustered into four subpopulations including CCs-Control, CCs-PCOS, oocyte- Control and oocyte-PCOS groups.

**G**, Significantly enriched GO terms (biological processes) of DEGs with upregulated expression in PCOS oocytes. **H,**Significantly enriched GO terms (biological processes) of DEGs with downregulated expression in PCOS oocytes.**I,** Signaling pathways enriched from DEGs with downregulated expression in PCOS oocytes. **G,** Signaling pathways enriched from DEGs with upregulated expression in PCOS oocytes.

**Figure S2:Significantly enriched function and signaling pathways in PCOS CCs**

**A,**Significantly enriched GO terms (biological processes) of DEGs with upregulated expression in PCOS CCs. **B,** Significantly enriched GO terms (biological processes) of DEGs with downregulated expression. **C,** Key signaling pathways enriched from DEGs with upregulated expression. **D,** Key signaling pathways enriched from DEGs with downregulated expression. **E,** Heatmap of PI3K-AKT signaling pathway upregulated in PCOS CCs .**F,** Heatmap of genes related to MAPK signaling pathway upregulated. **G,** Heatmap of Ras signaling pathway upregulated.

**Figure S3:Pathways involved in the Oocyte-CC interactions**

**A,** NOTCH signaling pathway involved in the Oocyte-CC interactions. Schematic diagram on left shows the relationship among these genes. Histograms on right show relative expression levels (log_2_ [TPM+2]) of ligands, receptors, and target genes in oocytes (n = 3, participants) and CCs (n = 2, participants). Red and blue bar represent PCOS and Control,respectively. Data represents mean ± SD. *p < 0.05, **p < 0.01, ***p < 0.001, ns, not significant. **B,** Gap junction involved in the Oocyte-CC interactions. Histograms on right show relative expression levels of related genes in oocytes and CCs. **C,** KITLG-KIT signaling pathway involved in the Oocyte-CC interactions. D, Relative expression levels of mitochondria-related genes in CCs from Controls and PCOS patients by qPCR analysis, *p < 0.05, **p < 0.01, ***p < 0.001.

**Figure S4:Expression pattern of TEs in oocytes and CCs from PCOS.**

**A,** Percentage of all differentially expressed TEs classified by repeat classes in PCOS oocytes. **B,** Scatter diagram showing differently expressed ERV elements in PCOS oocytes. Different color dots indicate different ERV elements and NS represents the ERV elements with no significant difference. **C,** Percentage of all differentially expressed TEs classified by repeat family in PCOS oocytes. **D,** Distribution of differentially expressed TEs in each chromosome.Red and blue dots representthe upregulated and downregulated TEs, respectively.**E,** Percentage of all differentially expressed TEs classified by repeat classes in PCOS CCs.

**Figure S5:Normality and variance equality test of clinical data.**

**A,** Normal Q-Q (Quantile- Quantile) plot of clinical characteristics and hormone levels**.** The abscissa represents the quantile of clinical data, and the ordinate represents the normal quantile. **B,** Equality of variance of clinical data was test by Levene’s Test.

**Figure S1**

**
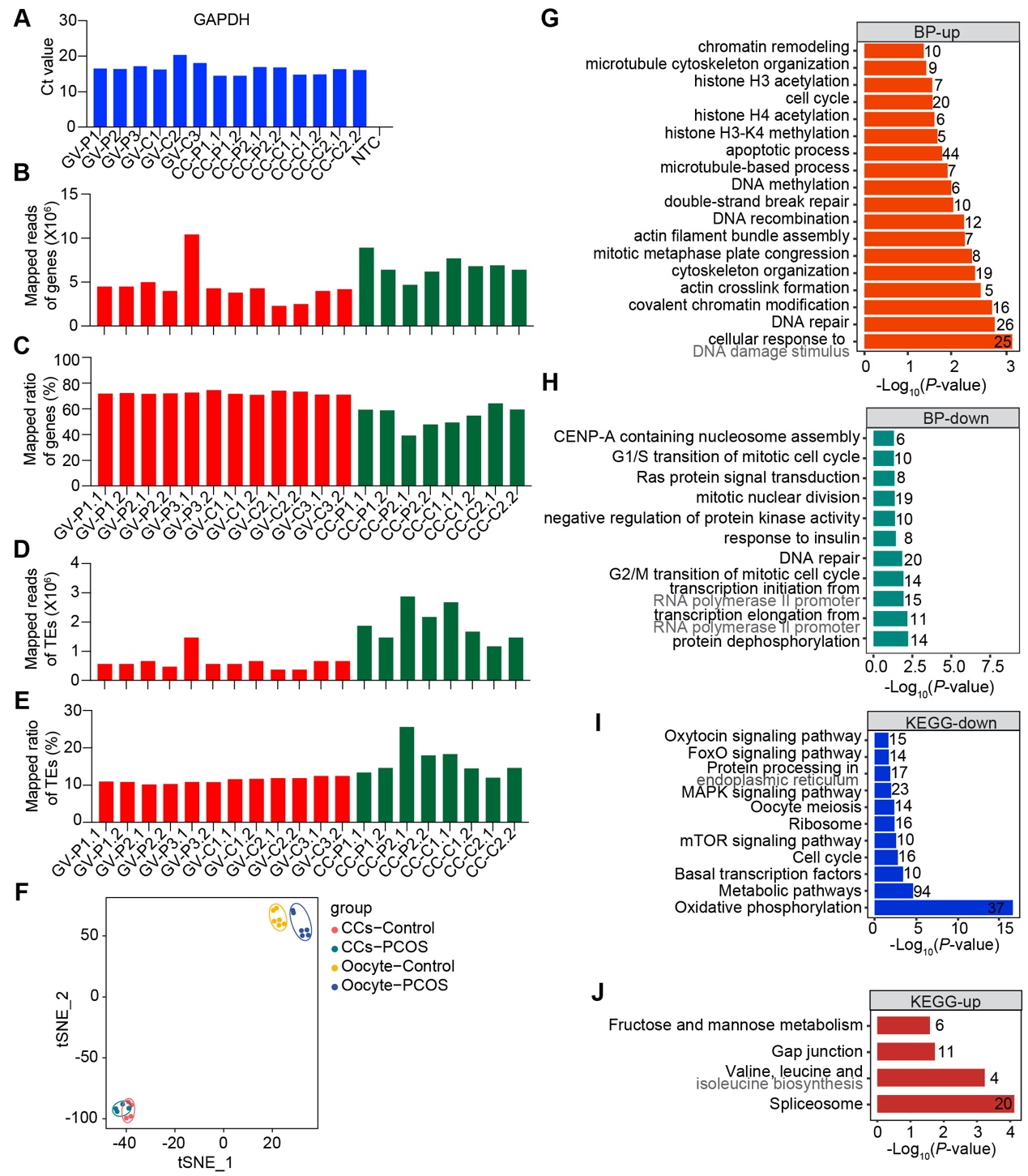
**

**Figure S2**

**
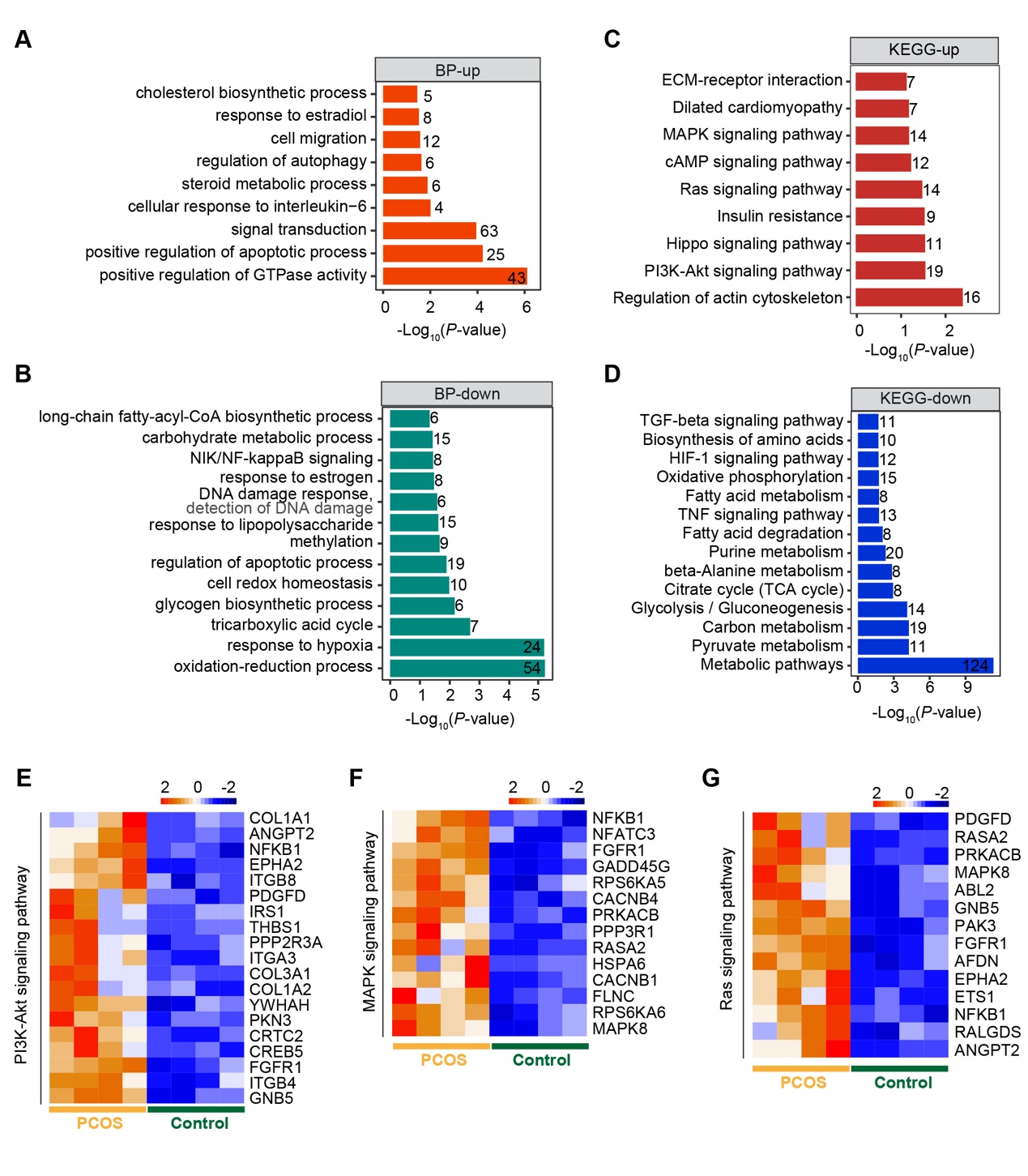
**

**Figure S3**

**
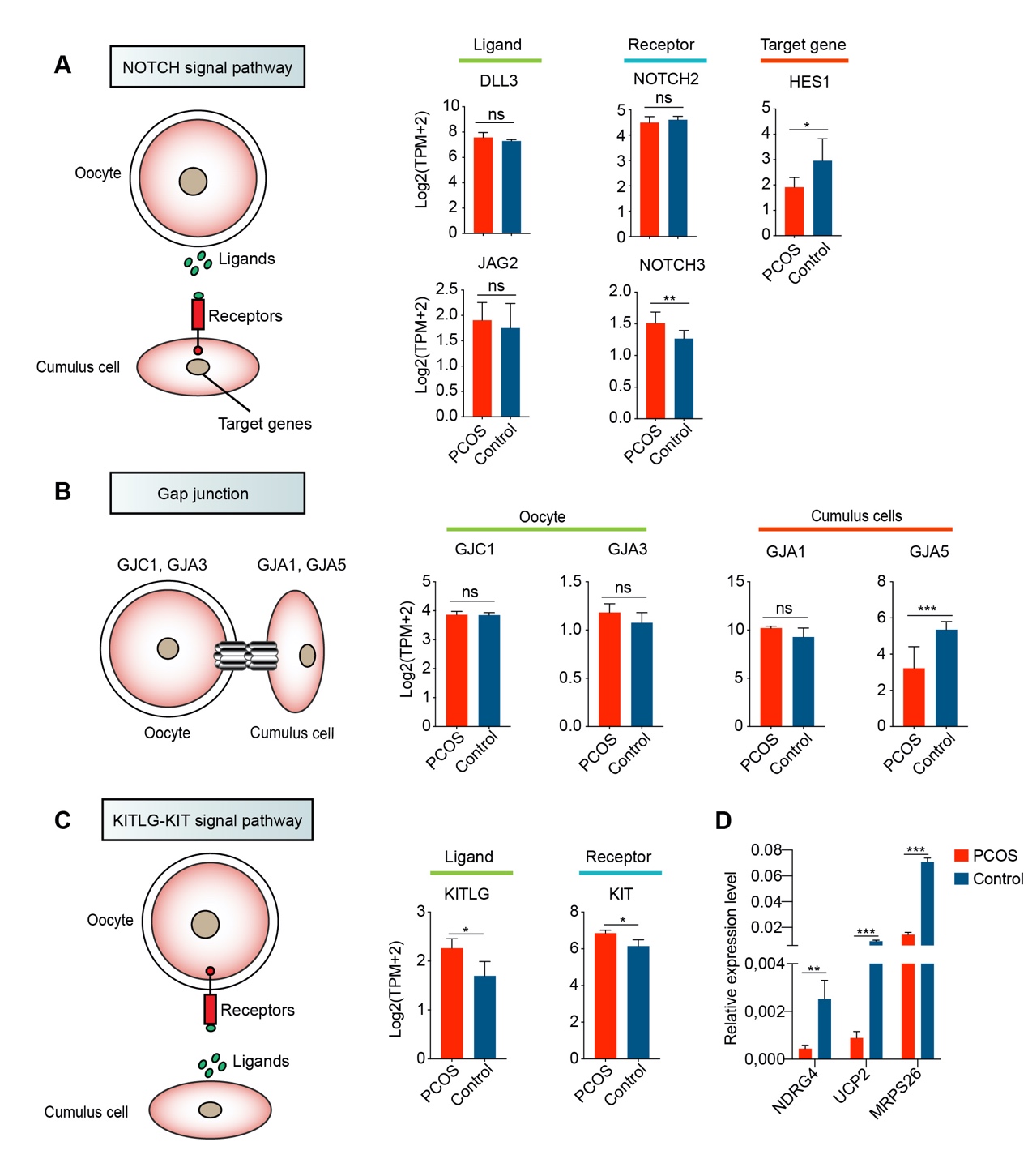
**

**Figure S4**

**
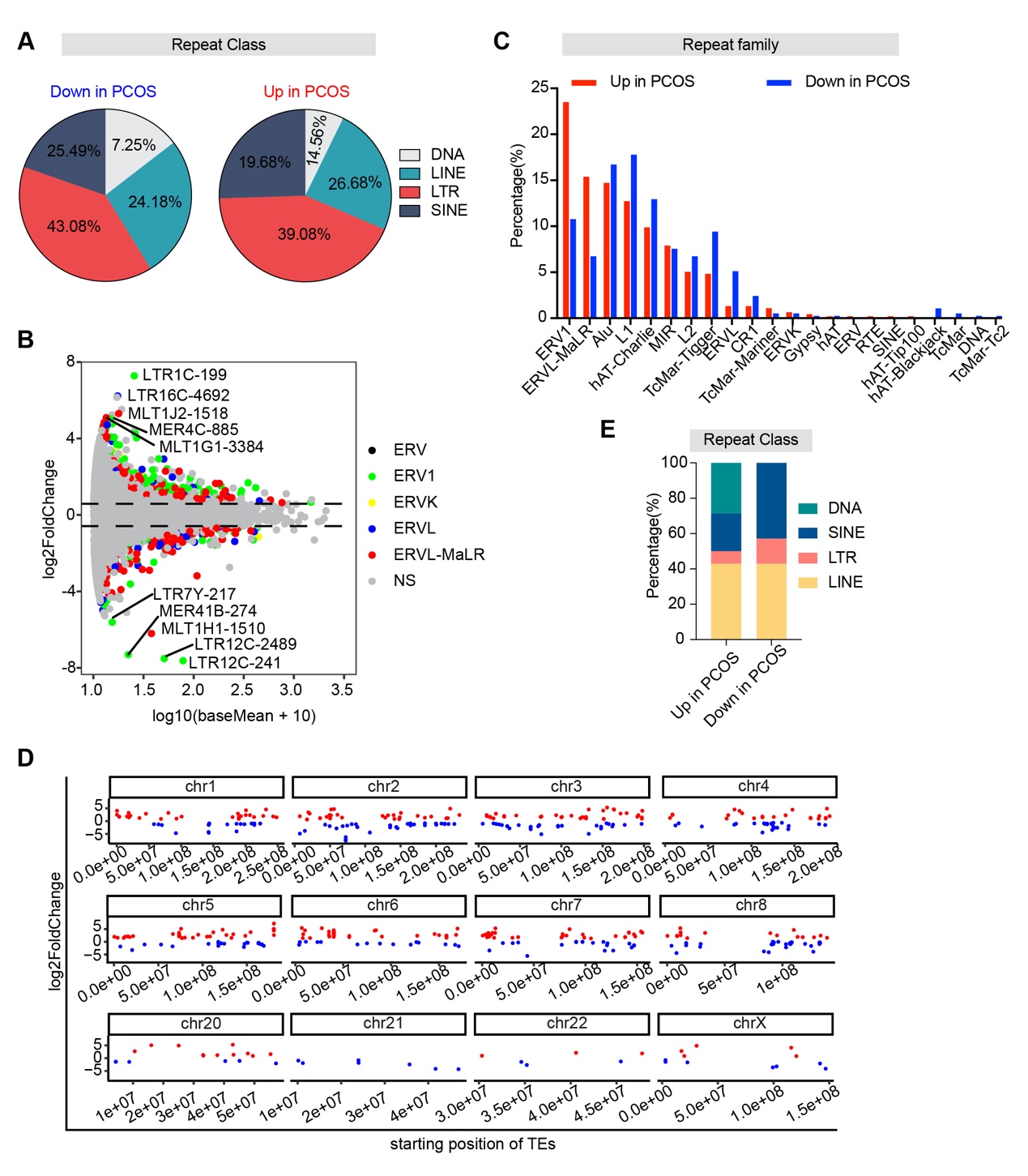
**

**Figure S5**

**
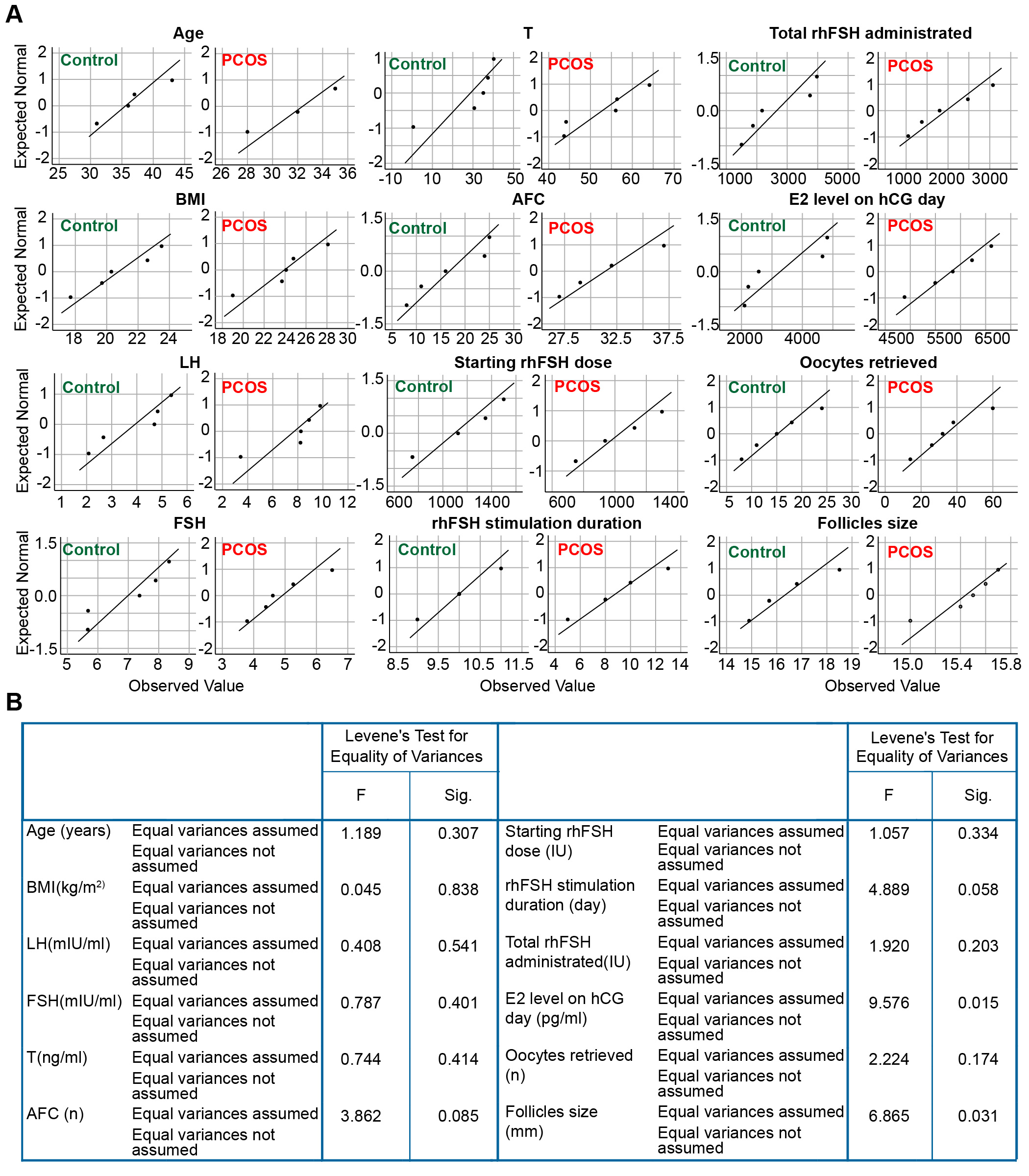
**
